# An Aqueous Extract of Radix Astragali, *Angelica sinensis*, and *Panax notoginseng* Is Effective in Preventing Diabetic Retinopathy

**DOI:** 10.1155/2013/578165

**Published:** 2013-04-08

**Authors:** Dehong Gao, Yijuan Guo, Xuejun Li, Xiumin Li, Zhipeng Li, Mei Xue, Zhimin Ou, Ming Liu, Mingxing Yang, Suhuan Liu, Shuyu Yang

**Affiliations:** ^1^Xiamen Diabetes Institute, The First Affiliated Hospital of Xiamen University, Xiamen 361003, China; ^2^Division of Endocrinology and Diabetes, The First Affiliated Hospital of Xiamen University, Xiamen 361003, China; ^3^Central Laboratory, The First Affiliated Hospital of Xiamen University, Xiamen 361003, China

## Abstract

Diabetic retinopathy (DR), in which inflammation has been implicated playing important roles, is one of the most common diabetes complications. Dang Gui Bu Xue Tang (DBT), an aqueous extract of Radix Astragali and Radix *Angelica sinensis*, is a classical prescription in Traditional Chinese Medicine for treating inflammation and ischemic diseases. Here, we investigated the effects of a modified recipe of DBT, with addition of *Panax notoginseng*, in treating diabetic retinopathy. An aqueous extract of Radix Astragali, Radix *Angelica sinensis*, and *Panax notoginseng* (RRP) was given to Goto-Kakizaki (GK) rats and streptozotocin-induced Sprague-Dawley (SD) rats. Leukostasis, vascular leakage, and acellular capillaries in retinal vasculature of animals were determined. Expression of retinal inflammatory biomarkers was assessed. We found that RRP reduced leukostasis, acellular capillaries, and vascular leakage compared to diabetic control rats. We also found that RRP decreased the expression of inflammatory factors including IL-1**β**, IL-6, TNF-**α**, NF-**κ**B, MCP-1, ICAM-1, or VCAM-1 in the retinas of GK rats and reversed high glucose-induced inhibition of endothelial cell migration and proliferation in vitro. We conclude that RRP has a potent effect in preventing the pathogenesis and/or progression of DR and thus may serve as a promising nontoxic therapeutic approach of DR.

## 1. Introduction

Diabetic retinopathy (DR) is a major cause of blindness among working-age adults in developed countries. Recent studies have identified inflammation as an important molecular mechanism in the development of DR [1]. During diabetes, hyperglycemia induces increased oxidative stress [2], inflammation [3], and vascular dysfunction through multiple cellular pathways and results in increased leukostasis [4, 5], vascular permeability [6], and formation of acellular capillaries [7]. Proinflammatory cytokines and chemokines activate the endothelium to increase expression of adhesion molecules and chemokines, by which leukocytes are mediated to attach to the vascular wall and transmigrate through the endothelium. Leukostasis has been found significantly increased in retinas of diabetic animals and might contribute to retinal vascular permeability and capillary nonperfusion in DR [8].

The Goto-Kakizaki (GK) rat, generated from glucose-intolerant Wistar rats, is considered to be a nonobese model of spontaneous type 2 diabetes with mild elevated glucose levels [9–11]. Regarding diabetic retinopathy, previous studies have reported that increased endothelial/pericyte ratio [12], subretinal accumulation of activated microglia/macrophages [13], increased vascular endothelial growth factor (VEGF) production in certain ocular tissue [14], and increased number of acellular capillaries [15], known as early histological changes of diabetic retinopathy, were confirmed in GK rats.

An aqueous extract of Radix *Angelica sinensis* and Radix Astragali, named Dang Gui Bu Xue Tang (DBT) and commonly used in treating ischemic ailments in traditional Chinese medicine, was modified here by addition of *Panax notoginseng* (the modified DBT recipe is expressed as RRP in this paper). In Chinese pin yin, the herb *Angelica sinensis* is pronounced as Dang Gui, Radix Astragali is pronounced as Huang Qi, and *Panax notoginseng *is pronounced as San Qi. Bu Xue refers to hematopoietic effects, and Tang refers to aqueous solution. Radix Astragali extract has been shown to have potent anti-inflammatory activity [16–22]. Radix *Angelica sinensis* has been used alone, or in combination with others, in the treatment of various inflammatory diseases [23–27]. *Panax notoginseng* saponins extracted from the roots of *Panax Notoginseng* are free radical-scavenger, with an antioxidant and anti-inflammatory property [28–36].

Diabetic retinopathy signs are broadly divided into nonproliferative and proliferative retinopathy. There is no clinical symptom in nonproliferative stage, while vision impairment appears when it progresses into proliferative stage. There is currently no effective intervention in preventing DR occurrence and progression; thus the present study was undertaken to investigate the effects of RRP in preventing and/or treating diabetic retinopathy in an animal model of type 2 diabetes. 

## 2. Materials and Methods

### 2.1. Standardization of RRP

RRP was provided by the Department of Pharmacy of the First Affiliated Hospital of Xiamen University, China. Identification of the major compounds in RRP was determined by high performance liquid chromatography (HPLC, Agilent 1200 HPLC system, Agilent, CA, USA). A Spherex C-18 analytical column (250 × 4.6 mm, 5.0 *μ*m, Phenomenex, CA, USA) was used with the mobile phase which consisted of 0.2% formic acid in water (A) and methanol (B). The mobile phase gradient elution was programmed as follows: 95–80% A (0–40 minutes), 65% B (40–50 minutes), 100% B (50–55 minutes), and 95% A (55–60 minutes). The column temperature was maintained at 35°C, the flow rate was set at 1 mL/minutes, and the sample injection volume was set at 10 *μ*L.

### 2.2. Animals and Treatments

Male Goto-Kakizaki (GK) rats and Wistar counterparts were purchased from Shanghai Experimental Animal center, Chinese Academy of Sciences (Shanghai, China). The rats were housed in temperature and humidity-controlled room, kept on a 12 h light/dark cycle and provided with unrestricted amount of rodent chow and water. At the age of 28 weeks, the rats were randomly allocated into four groups: normal vehicle treated control Wistar rats (Control), GK rats treated with vehicle (Diabetic, water was used as the vehicle in this study), GK rats treated with RRP at the dose of 4 g/kg body weight/d (RRP), and GK rats treated with Calcium Dobesilate at the dose of 200 mg/kg body weight/d (CD). Calcium Dobesilate, a therapeutic agent for prevention of diabetic retinopathy [37], was chosen here as a positive control. Vehicle, RRP, and CD were administered orally between 9 and 10 am once daily by gavage for 12 weeks. Body weight and blood glucose measurements were performed twice weekly. Glucose measurements were taken from the tail vein and measured using OneTouch Ultra Blood Glucose Meter (LifeScan, USA). Triglyceride (TG) and total cholesterol (TC) were assayed by enzymatic methods with automatic multichannel chemical analyzer (Hitachi 7450, Hitachi Corp., Tokyo, Japan). Type 1 diabetes was induced by a single intraperitoneal injection of streptozotocin (STZ) (60 mg/kg) in 8-week-old male Sprague-Dawley (SD) rats. Age-matched control rats received an equal volume of vehicle (0.01 M citrate buffer, pH 4.5). 48 h after STZ injection, the blood glucose level was measured from the tail vein. Rats with a blood glucose level over 20 mmol/L were considered to be diabetic. The animals were then divided into three groups: (1) normal SD rats (control, *n* = 10), (2) diabetic rats (Diabetic, *n* = 10), and (3) diabetic rats with RRP treatment (4 g/kg body weight/d) (RRP,  *n* = 10). All animal experiments were approved by Xiamen University Animal Care and Use Committee.

### 2.3. Measurement of Retinal Endothelial Permeability

Retinal endothelial permeability was measured using the Evans blue (EB) dye injection method as previously described [38] with minor modifications. Briefly, EB (Sigma, St. Louis, MO) was dissolved in saline (30 mg/mL), filtered, and injected through the tail vein at a dosage of 45 mg/kg within 10 seconds. After the dye had circulated for 2 hours, the rats were anesthetized with pentobarbital (40 mg/kg body weight), the chest cavity was opened, and cardiac perfusion was performed via the left ventricle with 1% paraformaldehyde in citrate buffer (0.05 M, pH 3.5) under a constant pressure of 120 mm Hg. Immediately after perfusion, the retinas were carefully dissected under an operating microscope. After retinas were fully dried at 4°C then the weights of them were measured, EB dye was extracted by incubating each sample in 150 *μ*L formamide for 18 hours at 70°C. The extract was centrifuged at 14,000 rpm for 60 minutes at 25°C. Absorbance was measured using 100 *μ*L of the supernatant at 620 nm and 740 nm. The concentration of EB (ng/mg protein) in the extracts was calculated from a standard curve and normalized by the weight of the dry retina (mg).

### 2.4. Visualization and Quantification of Retinal Vascular Leakage Using FITC-Labeled BSA

Anesthetized SD rats received tail vein injections of fluorescein isothiocyanate-BSA (FITC-BSA, 100 mg/kg) (Sigma-Aldrich). After 20 min, rats were sacrificed and eyes were removed and immediately fixed in 4% paraformaldehyde for 30 min. Retinas were dissected, flatly mounted onto a glass slide, and imaged by fluorescent microscopy (IX71, Olympus, Tokyo, Japan). A computer-assisted method was used to quantify leakage using Image Pro 6.0 software. In this procedure, the intensity of fluorescence in nonleakage areas was used as background fluorescence. After deduction of background signals, the total intensity of fluorescence contributed by the leaked FITC-BSA was used to represent the leakage.

### 2.5. Quantification of Retinal Acellular Capillaries

 To analyze the retinal vasculature for acellular capillaries, retinal vasculature was isolated using the trypsin digest method [39] with slight modifications. Briefly, 10% buffered formalin-fixed retinas were dissected after enucleation, washed in PBS overnight, and incubated with 3% trypsin (Invitrogen-Gibco, Grand Island, NY) at 37°C for 2-3 h with gentle shaking. Retinal tissues were brushed away in water, and the isolated vasculature was mounted on glass slides and stained with periodic acid-Schiff (Sigma-Aldrich, St. Louis, MO) and hematoxylin. Acellular capillaries were counted in six field areas from each quadrant of the retina using 40x objective lenses and expressed as the total number of acellular vessels per square millimeter of retina area.

### 2.6. Quantification of Retinal Leukostasis

Quantification of leukostasis was performed using fluorescein-isothiocyanate- (FITC-) conjugated concanavalin A (ConA) as described previously [4]. The chest cavity of each deeply anesthetized rat was carefully opened and a perfusion needle was inserted into the left ventricle. After cutting the right atrium, the animals were immediately perfused with 500 mL of PBS per kg body weight and heparin (0.1 mg/mL) to wash out nonadherent blood cells. FITC-conjugated ConA (20 *μ*g/mL in PBS; pH 7.4; 5 mg/kg body weight; Sigma-Aldrich, St. Louis, MO) was then perfused to label adherent leukocytes and vascular endothelial cells. Residual unbound ConA was flushed by PBS perfusion. Eyes were removed and fixed in 4% paraformaldehyde for 1 h. The retinas were carefully dissected and flat mounted. Images of retinas were observed by confocal microscopy (Leica TCS SP5, Leica Microsystems, Wetzlar, Germany), and the total number of adherent leukocytes within the vessels of each retina was counted.

### 2.7. ELISA Assay

Activation of the transcription factor NF-*κ*B was determined in the retina by ELISA kit from Active Motif (Carlsbad, CA) based on the principle that only the active form of NF-*κ*B in the sample binds to oligonucleotide containing the NF-*κ*B consensus site (5′-GGG ACT TTC C-3′) which is immobilized on the microtiter plate. The primary antibody against the p65 subunit of NF-*κ*B was used in the assay and the secondary antibody used is conjugated to horseradish peroxidase. Retina was homogenized in the lysis buffer (as provided by the manufacturer), and after removing the cell debris, 10 *μ*g protein was used for the ELISA [40]. Retinal VEGF levels were quantified by ELISA using a kit from R&D Systems, MN. The samples were incubated for 2 hours and washed out, and then the plate was incubated with rat VEGF conjugate. This assay was sensitive to the concentration of VEGF as low as 15 ng/mL.

### 2.8. Western Blot Analysis

Retinas were dissected and sonicated in a lysis buffer containing 50 mmol/L Tris (pH 7.6), 150 mmol/L NaCl, 5 mmol/L EDTA, 1% Triton X-100, 0.1% SDS, 0.5% deoxycholate, and a protease inhibitor cocktail (Roche Molecular Biochemicals, Indianapolis, IN). The lysate was centrifuged at 14,000 rpm for 10 min at 4°C. Supernatants were collected and determined the protein concentrations using the Bradford method. Retinal proteins were then separated by SDS-polyacrylamide gel electrophoresis and transferred to polyvinylidene fluoride membrane. After blocking, membranes were blotted overnight at 4°C with anti-VEGF (1 : 500), antioccludin (1 : 500) (Santa Cruz, CA), and anti-*β*-actin (1 : 1000) antibodies (Sigma-Aldrich). Horseradish peroxidase-linked anti-rabbit or mouse (1 : 10,000) (Santa Cruz, CA) was used for secondary detection. Immunoreactivity was visualized using an ECL kit (Amersham Pharmacia Biotech, Piscataway, NJ) and Kodak X-OMAT film (Eastman Kodak Co., Rochester, NY). Band intensities were quantified using a gel documentation system and Quantity One software (Bio-Rad).

### 2.9. Cell Culture and Treatment

EA.hy926, an endothelial cell line, were starved in DMEM containing 0.5% FBS for 24 h and then cultured in DMEM culture medium with either 5.5 (N), 25 mM d-glucose (H), or 25 mM d-glucose with 1 h RRP before treatment for 24 h. This medium was supplemented with 10% FBS, 1% l-glutamine, 2% HAT, and 1% penicillin/streptomycin in a humidified atmosphere of 5% CO_2_ at 37°C.

### 2.10. Cell Viability and Migration Assay

CCK8 assay was used to measure cell viability. In brief, EA.hy926 cells were seeded into 96-well plates at a density of 1000 cells/well in 100 *μ*L DMEM culture medium (5.5 mM d-glucose) and allowed to adhere overnight and then cultured in culture medium with either 5.5 mM (N), 25 mM (H), or 25 mM d-glucose pretreated with RRP for 24 hours. After that, 10 *μ*L of CCK8 was added to each well for another 2 h at 37°C and the absorbance measured at 450 nm. 

The role of high glucose and/or RRP on the migration of EA.hy926 cells was analyzed by cell scratch assay. The cells were plated at 70,000 cells/well in a 12-well plate with DMEM (5.5 mM d-glucose) till cells become 80% confluent. The cells was scratched once using a sterile 1 mL pipette tip and washed twice with complete medium. DMEM with either 5.5 mM (N), 25 mM (H), or 25 mM d-glucose pretreated with RRP was then added in complete medium, and the incubation continued at 37°C. An image was made at 0 and 24 h after the addition of high glucose and/or RRP using a microscopy (IX71, Olympus, Tokyo, Japan). The width of the gap after 24 h was measured and the extent of migration was calculated. 

### 2.11. Real-Time Polymerase Chain Reaction (PCR)

Total RNA was extracted using RNA simple Total RNA Kit (Tiangen Biotech, China). Reverse transcription of total RNA to cDNA was carried out with primeScript RT reagent kit with gDNA eraser (TaKaRa, China) in a MyCycler Thermal Cycler (Bio-Rad, USA) following the manufacturer's instruction. Real-time quantitative PCR was performed with the SYBR Premix Ex Taq real-time PCR kit (Takara, Dalian, China) in a Light Cycler 480 System (Roche). The following primers were used in this study: Tumor necrosis factor-*α* (TNF-*α*) sense, 5′-ACA CCA TGA GCA CGG AAA GC-3′; antisense, 5′-CCG CCA CGA GCA GGA A-3′; Interleukin-1*β* (IL-1*β*) sense, 5′-AAT GGA CAG AAC ATA AGC CAA CA-3′; antisense, 5′-CCC AAG GCC ACA GGG AT-3′; Interleukin-6 (IL-6) sense, 5′-GTT GCC TTC TTG GGA CTG ATG-3′; antisense, 5′-ATA CTG GTC TGT TGT GGG TGG T-3′; nuclear transcription factor-*κ*B (NF-*κ*B)/p65 sense, 5′-AGA AGC GAG ACC TGG AGC AA-3′; antisense, 5′-CGG ACC GCA TTC AAG TCA TAG-3′; VEGF sense, 5′-ACA GGG AAG ACA ATG GGA TGA-3′; antisense, 5′-GGG CCA GGG ATG GGT TT-3′; monocyte chemotactic proteins-1 (MCP-1) sense, 5′-AAT GGG TCC AGA AGT ACA TTA GAA A-3′; antisense, 5′-GGT GCT GAA GTC CTT AGG GTT G-3′; intercellular adhesion molecule-1 (ICAM-1) sense, 5′-CGG GTT TGG GCT TCT CC-3′; antisense, 5′-GCC ACT GCT CGT CCA CAT AG-3′; vascular cell adhesion molecule-1 (VCAM-1) sense, 5′-ATC TTC GGA GCC TCA ACG G-3′; antisense, 5′-CCA ATC TGA GCG AGC GTT T-3′; *β*-actin sense, 5′-CCC ATC TAT GAG GGT TAC GC-3′; antisense, 5′-TTT AAT GTC ACG CAC GAT TTC-3′. To determine the relative expression levels, the threshold cycle (Ct) values of target genes were normalized with *β*-actin of the same sample and expressed as relative to controls.

### 2.12. Statistical Analysis

All results were presented as the mean ± SD. Statistical evaluation of the results was performed using a one-way analysis of variance (ANOVA) followed by Tukey's multiple comparison test using GraphPad Prism 5.0 software (GraphPad, CA, USA). Values of *P* < 0.05 were considered statistically significant.

## 3. Results

### 3.1. HPLC Analysis of RRP

**Figure 1:**
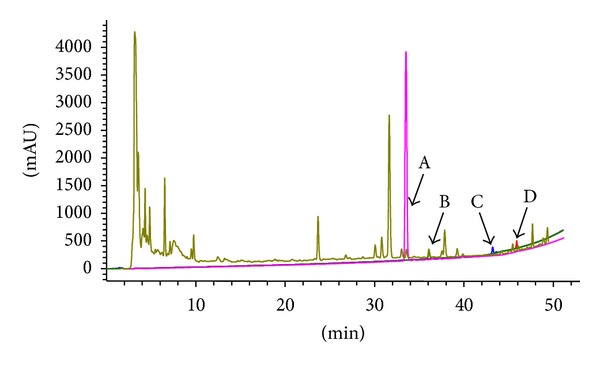
Chemical standardization of RRP by HPLC fingerprint analysis. In the HPLC fingerprint at an absorbance of 203 nm, the peaks corresponding to calycosin (A), ginsenoside Rg1 (B), ligustilide (C), and ginsenoside Rb1 (D) were identified.

 In order to standardize the herbal extract chemically, we performed HPLC analysis.  Figure 1 shows a typical HPLC fingerprint of RRP, in which major peaks were identified by comparing both the retention times of RRP and reference standards; 4 compounds (A: calycosin; B: ginsenoside Rg1; C: ligustilide; D: ginsenoside Rb1) in RRP were well identified. 

### 3.2. RRP Has No Effect on Body Weight, Blood Glucose, and TG and TC Levels

After 12-week treatment, body weight and TG showed no difference among groups. As shown in Table 1, all GK rats developed hyperglycemia and hypercholesterolemia (TC) compared to the normal control Wistar rats (*P* < 0.05), but no significant differences were found in all four GK rats groups, suggesting that neither CD nor RRP has any effect in restoring the disordered glucose and lipid metabolism in GK rats.

### 3.3. RRP Attenuated Retinal Vascular Permeability

**Figure 2:**
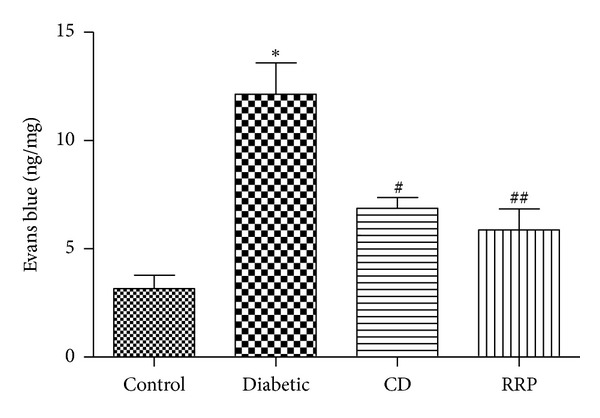
Vascular permeability in the retinas from normal Wistar rats (Control), GK rats treated with vehicle (Diabetic), GK rats treated with CD (CD), and GK rats treated with RRP (RRP) was measured with Evans blue as a tracer. Evans blue was normalized by total protein concentration in the tissue. Permeability is expressed as ng of dye per mg of protein in the tissue. All data were expressed as mean ± SD (*n* = 6). *P < 0.05 versus Control group; ^#^ *P* < 0.05 versus Diabetic group; ^##^ *P* < 0.01 versus Diabetic group.

Blood-retinal barrier (BRB) breakdown is a hallmark of diabetic retinopathy, evidenced by increased blood vessel permeability. The retinal blood vessel permeability in Diabetic group significantly increased (12.1 ± 4.5 ng/mg) compared with that in control group (3.2 ± 1.4 ng/mg) (*P* < 0.001) showing an impaired BRB in diabetes. CD treatment significantly decreased retinal vascular permeability (6.9 ± 1.0 ng/mg, *P* < 0.05), whereas RRP reversed the retinal vascular permeability to a further extent (5.9 ± 2.4 ng/mg, *P* < 0.01) (Figure 2). 

**Figure 3:**
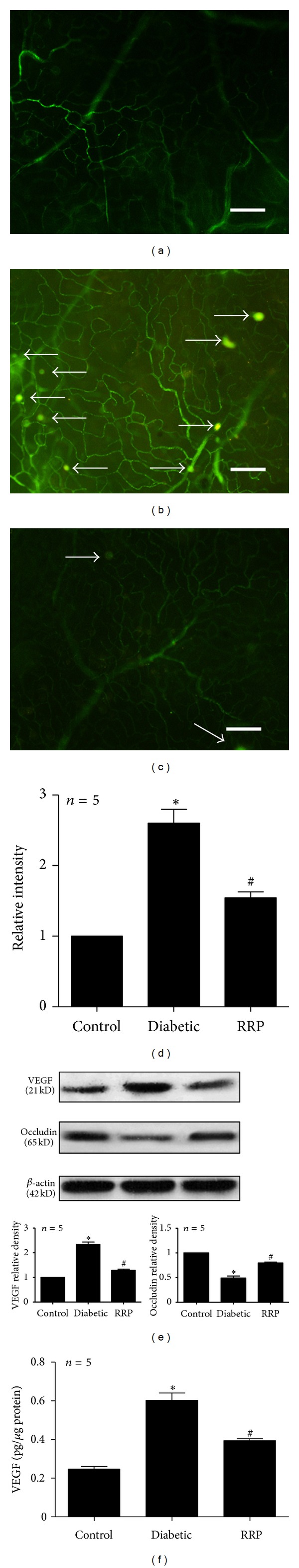
(a–d) Analysis of retinal vascular leakage in the retinas from (a) normal control rats (Control), (b) STZ-induced diabetic rats (Diabetic), and (c) STZ-induced diabetic rats treated with RRP (RRP) 3 weeks after the onset of diabetes using FITC-labeled albumin. Microscopic images of retinal flat mounts (a–c) showing fluorescently labeled albumin (arrowheads) in retinas of SD rats. (d) Quantification of the FITC-labeled albumin from (a), (b), and (c). Scale bars represent 100 *μ*m. (e) Western blot analysis of retinal VEGF and occludin expression in Control, Diabetic, and RRP groups. (f) VEGF concentrations were measured in the retina of rats in Control, Diabetic, and RRP groups using an ELISA kit from R&D Systems. All data were expressed as mean ± SD (*n* = 5). *P < 0.05 versus normal SD rats; ^#^ *P* < 0.05 versus Diabetic SD rats.

To further investigate the breakdown of the blood-retinal barrier, we measured vascular leakage in a group of STZ-induced diabetic rats using FITC-labeled albumin. Vascular leakage was visualized and quantified in diabetic SD rats 3 weeks after inducing diabetes (Figures 3(a)–3(d)). Similar to GK rats, compared with the control SD rats, diabetic SD rats had more leaked FITC-labeled albumin (Figure 3(b)) in their retina, and RRP treatment significantly decreased the retinal vascular leakage (Figure 3(c)). Computer-assisted quantitative analysis demonstrated a similar level of FITC-labeled albumin leakage in RRP treated diabetic rats and control SD rats controls (Figure 3(d)), whereas the FITC-labeled albumin leakage in diabetic rats was significantly higher. 

To determine the mechanism of RRP effect in diabetes-induced vascular leakage, we examined the protein expression of VEGF and occludin (important tight junction protein). Western blot analysis of retinas from control SD rats showed significantly increased VEGF level and decreased occludin level in diabetic SD rats' retinas, and RRP treatment greatly reversed both these changes (Figure 3(e)). Retinal VEGF protein level was confirmed by ELISA assay (Figure 3(f)) which also showed that the level of retinal VEGF protein increased in diabetic rats group compared to control, and was reduced by RRP treatment.

Given the fact that there was no change of blood glucose levels, the restored retinal vascular permeability by RRP should be attributed to the improved local microenvironment.

### 3.4. RRP Treatment Prevented Retinal Capillary Vasoregression

**Figure 4:**
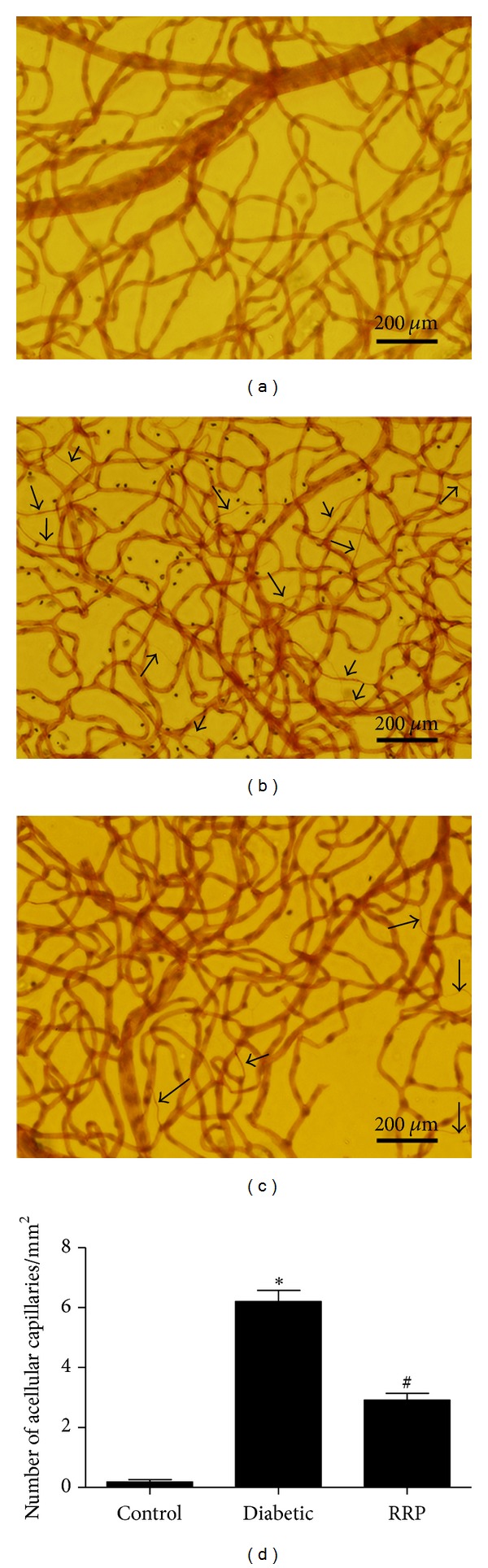
Analysis of acellular capillaries (arrowheads) in retinas from (a) normal Wistar rats (Control), (b) GK rats treated with vehicle (Diabetic), and (c) GK rats treated with RRP (RRP). (d) Quantification of the number of acellular capillaries from (a), (b), and (c). Scale bar represents 200 *μ*m. All data were expressed as mean ± SD (*n* = 3). *P < 0.001 versus Control rats; ^#^ *P* < 0.001 versus Diabetic rats.

 To further assess the effect of RRP on retinal vascular lesions in diabetic GK rats, we quantified the number of acellular capillaries, a sign of retinal capillary vasoregression, by trypsin digestion assay. The number of retinal acellular capillaries of diabetic rats was significantly increased about 6-fold compared to the control rats (Figures 4(a) and 4(b)) (*P* < 0.001), suggesting a damaged and degenerated microcirculation surroundings. RRP treatment largely reduced this vascular lesion by 52.6% (*P* < 0.001) (Figures 4(c) and 4(d)).

### 3.5. RRP Decreased Retinal Leukostasis

**Figure 5:**
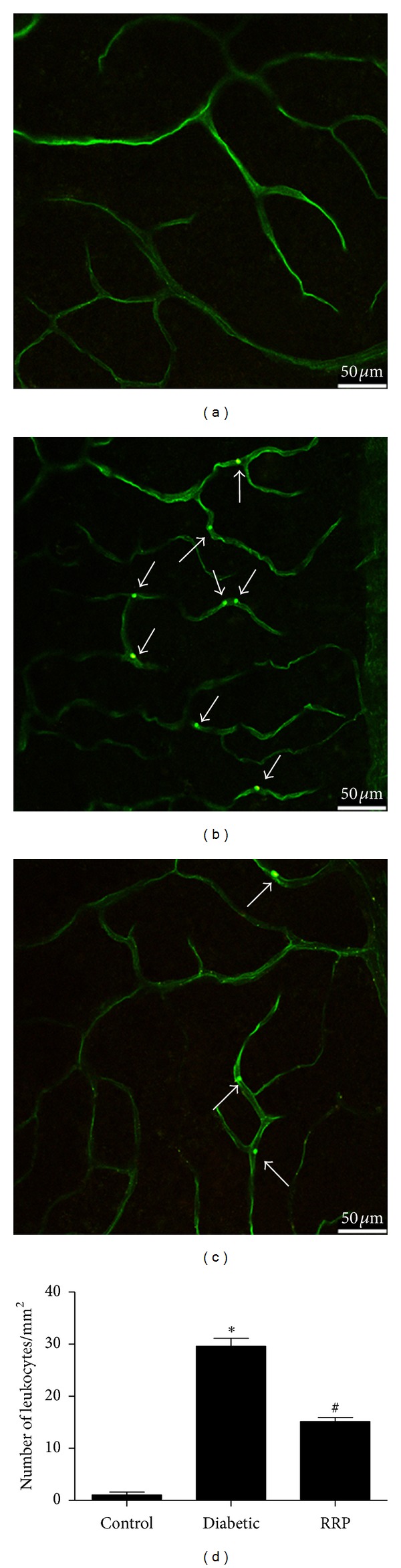
Analysis of leukocyte adhesion (arrowheads) in retinas from (a) normal Wistar rats (Control), (b) GK rats treated with vehicle (Diabetic), and (c) GK rats treated with RRP (RRP). (d) Quantification of the number of leukocytes from (a), (b), and (c). Scale bar represents 50 *μ*m. All data were expressed as mean ± SD (*n* = 3). *P < 0.001 versus Control group; ^#^ *P* < 0.001 versus Diabetic group.

It has been shown that diabetic retinal vascular leakage and nonperfusion are temporally and spatially associated with retinal leukocyte stasis (leukostasis) in the rat model of streptozotocin-induced diabetes [41]. Leukostasis, a major parameter for inflammation and early pathologic changes in DR, was labeled with FITC-conjugated ConA. The number of adherent leukocytes in the retinal microvasculature of Control Wistar rats was negligible (Figure 5(a)), while diabetes caused a significant increase of leukocytes adhesion to the endothelia cells (Figure 4(b)) (*P* < 0.001). Twelve-week administration of RRP significantly decreased retinal leukostasis by 44.8% compared to Diabetic group (*P* < 0.001) (Figures 5(c) and 5(d)).

### 3.6. RRP Decreased the Gene Expression of Inflammatory Cytokines In Vivo and In Vitro

**Figure 6:**
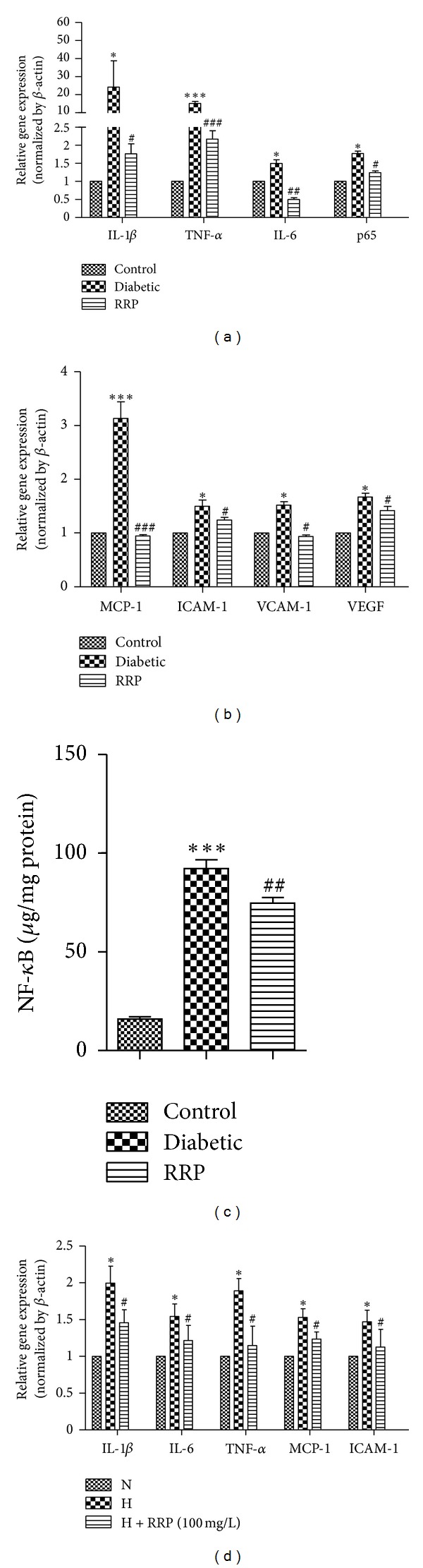
(a, b) Real-time PCR analysis of gene expression of pro-inflammatory markers and (c) ELISA assay detection of NF-*κ*B activation in the retinas from normal Wistar rat (Control), GK rats treated with vehicle (Diabetic), and GK rat treated with RRP (RRP) (*n* = 3). *P < 0.05, and ***P < 0.001 versus Control group; ^#^ *P* < 0.05, ^##^ *P* < 0.01, ^###^ *P* < 0.001 versus Diabetic group. (d) Real-time PCR analysis of gene expression of pro-inflammatory markers in EA.hy926 cells cultured in DMEM medium with either 5.5 (N), 25 mM glucose (H), or 25 mM glucose before treatment with RRP (dissolved in dimethyl sulfoxide) 1 h ago for 24 h after starving in DMEM containing 5.5 mM glucose for 24 h. *P < 0.05 versus N group; ^#^ *P* < 0.05 versus H group (*n* = 5). All data were expressed as mean ± SD.

To verify the effect of RRP on diabetic retinal inflammation, we examined the gene expression of proinflammatory markers, adhesion molecules, and chemokines. All inflammatory mediators were expressed at very low levels in the retina of control rats (Figure 6). In Diabetic group, retinal mRNA levels of IL-1*β*, IL-6, TNF-*α*, NF-*κ*B p65, VEGF, MCP-1, ICAM-1, and VCAM-1 were significantly increased (1.5- to 35-fold) compared to Control rats (*P* < 0.05) (Figures 6(a) and 6(b)). These increases were restored to control values in rats treated with RRP. The activation of NF-*κ*B in Diabetic group was significantly increased compared to Control group (*P* < 0.001) (Figure 6(c)) and decreased significantly by RRP treatment (*P* < 0.01). RRP inhibition of inflammatory cytokines was reproduced in vitro in cultured endothelial cell line—EA.hy926 cells. The gene expression of inflammatory cytokines and adhesion molecules increased dramatically after the cells were incubated in high glucose (25 mm/L) medium for 24 h, while RRP treatment significantly blocked this deleterious effect (Figure 6(d)).

### 3.7. RRP Reverses High Glucose-Induced Inhibition of Endothelial Cell Migration and Proliferation

**Figure 7:**
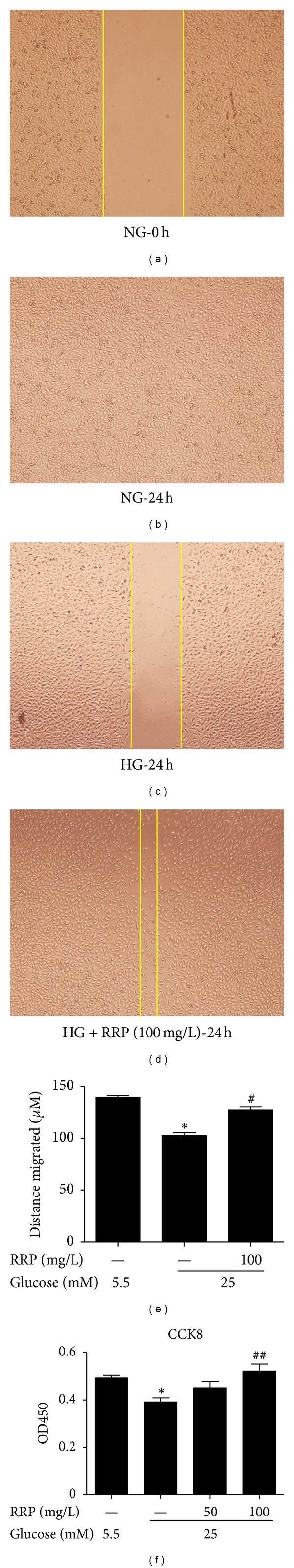
EA.hy926 cells were incubated with 5.5 mM D-glucose (NG), 25 mM D-glucose (HG), and HG treated with RRP for 24 h. (a–e) RRP reversed high glucose induced suppression of EA.hy926 cells migration. The width of the gap after 24 h was measured and subtracted from that at 0 h to quantify the distance the cells migrated. Yellow lines indicate distance between EA.hy926 cells on both sides of wound. (f) RRP reversed high glucose induced suppression of EA.hy926 cells proliferation. Measurements were made by CCK8 assay. All data were expressed as mean ± SD (*n* = 5). *P < 0.05 versus normal glucose group; ^#^ *P* < 0.05 versus high glucose group.

In wound-healing assays in vitro, high glucose markedly attenuated EA.hy926 cells migration, and this effect was reversed by RRP addition (Figures 7(a)–7(c)). High glucose decreased the viability of EA.hy926 cells as measured by CCK8 assay and this effect was reversed by RRP (Figure 7(d)).

## 4. Discussion

Dang Gui Bu Xue Tang, an aqueous extracts of Radix Astragali and Radix *Angelica sinensis*, a commonly used prescription in treating ischemic ailments in traditional Chinese medicine (TCM), was modified here by addition of *Panax notoginseng* (RRP) and was investigated in treating diabetic retinopathy. All these herbal medicines have anti-inflammatory properties and are frequently used for the treatment of diabetes and diabetic complications in TCM [26, 42]. We showed here that RRP treatment prevented leukocyte adherent to the vascular wall, attenuated vascular leakage, inhibited formation of acellular capillaries, the three early signature pathologies of diabetic retinopathy, thus ameliorated the retina damage and prevented DR progression in rat model of diabetes. RRP protection from diabetic retinopathy may be via inhibition of inflammatory response and improvement of microcirculation in the retina. 

DR is a low-grade chronic inflammatory condition [43]. Previous studies have shown that anti-inflammatory drugs can prevent early DR [44, 45]. Radix Astragali, Radix *Angelica sinensis*, and *Panax notoginseng* have been shown inhibit proinflammatory factors or chemokines expression such as IL-1*β*, IL-6, TNF-*α*, VEGF, MCP-1, ICAM-1 or VCAM-1 [18, 23–25, 35, 36]. Here we confirmed that RRP suppressed the gene expression of a series of proinflammatory cytokines which may consequently result in decreased inflammation in retina of diabetic rats (Figure 6). Proinflammatory cytokines (such as TNF-*α* and interleukins) and chemokines (such as MCP-1) activate endothelium to increase expression of adhesion molecules (such as ICAM-1, VCAM-1) and chemokines, by which leukocytes/monocytes were mediated to attach to the vessel wall and transmigrate through the endothelium [1]. ICAM-1 blockade prevents diabetic retinal leukostasis and vascular leakage [41]. We had speculated that RRP may protect retina from leukostasis via blockade of the inflammation and subsequent activation of leukocyte/monocyte adhesion process. After 12-week administration, we observed that RRP suppressed inflammatory gene expression and NF-*κ*B activation and efficaciously reversed diabetes-induced elevation of ICAM-1 and VCAM-1 levels. Increased leukostasis is an early event in DR [5] and is proposed lead to BRB breakdown, endothelial cell lesion, and capillary loss [46]. In this study, we observed that the number of leukocytes/monocytes which adhered to the retinal vascular endothelium was increased in diabetic GK rats; accordingly, vascular permeability and the number of acellular capillaries increased dramatically in diabetic GK rats. RRP treatment inhibited the leukostasis in retinal vasculature (Figure 5), which may be benefited from the suppressed expression of ICAM-1 and VCAM-1 molecules. Alteration of the inner BRB is the hallmark of DR and breakdown of the BRB appears early in the progression of retinopathy in GK rats [47], as well as in diabetic patients [48, 49]. In consistent with the attenuated leukostasis, we found that, compared with untreated Diabetic group, 12 weeks application of RRP significantly reduced the retinal vascular permeability of diabetic GK rats (Figure 2) and inhibited the formation of acellular capillaries (Figure 4). Furthermore, 3-week application of RRP greatly reduced the retinal vascular leakage in STZ-induced diabetic rats (Figure 3), suggesting an overall protection of RRP in preventing retina damage in diabetes. We did not see obvious effect of RRP on body weight, blood glucose, and TG or TC levels (Table 1) while the improvement of retinopathy was clear, implying that the hyperglycemia-induced local environment change especially inflammation plays more important roles in the pathogenesis of retinopathy than the systemic hyperglycemia. It has been shown retinal inflammatory mediators are principal in the resistance of retinopathy to arrest after cessation of hyperglycemia, and proinflammatory cytokines and adhesion molecules remained high after good glycemic control for at least six months that has followed six months of poor glycemic control [50]. Reversal of hyperglycemia fails to provide any significant effect on the development of histopathology that is characteristic of DR [51]. Various studies have demonstrated that inhibition of pro-inflammatory cytokines (such as IL-1*β*, TNF-*α*, and VEGF), chemokines (such as MCP-1), or adhesion molecules (such as ICAM-1) can reduce diabetes-induced leukostasis [3, 41, 44], degeneration of retinal capillaries [52], or BRB breakdown [53]. Thus, our results suggest that the antiretinopathy effect of RRP may be unrelated to lowering blood glucose, but it is due to the inhibition of inflammation in the retinas of GK rats. In addition to the in vivo study in diabetic GK rats, we also confirmed the RRP inhibition effect of inflammatory cytokines in cultured endothelial cells.

Endothelial cell loss via apoptosis was the early pathological change in DR; we showed here that RRP reversed the inhibited cell migration and decreased cell death induced by high glucose (Figure 7) in cultured EA.hy926 cells, suggesting a protective effect of RRP in preventing early endothelial cell death [43].

In summary, our results demonstrated that RRP had an inhibitive effect in the development and/or progression of DR in GK rats. RRP prevention of retinopathy may be associated with the inhibition of leukostasis and inflammation. Diabetic patients usually go to see the ophthalmologist only after the visual complications have already occurred, when there is no much ideal intervention available to reverse or eliminate the damage. With strong effects in ameliorating feature characteristics of DR-increased permeability, leukostasis, and acellular capillaries, RRP may serve as a prevention and/or intervention strategies in managing DR. 

## Figures and Tables

**Table 1:** Metabolic and physical parameters.

Group	Body weight (g)	Blood glucose (mmol/L)	Cholesterol (mmol/L)	Triglyceride (mmol/L)
Control	409.0 ± 31.2	6.272 ± 0.856	1.486 ± 0.143	0.953 ± 0.242
Diabetic	411.0 ± 47.3	19.718 ± 3.897*	2.527 ± 0.214*	1.003 ± 0.445
CD	409.8 ± 32.1	21.798 ± 2.976*	2.693 ± 0.260*	0.910 ± 0.454
RRP	411.2 ± 33.0	18.510 ± 7.319*	2.422 ± 0.108*	0.982 ± 0.353

All data were expressed as mean ± SD. *P < 0.05 versus Control group.
